# Hypofractionated radiotherapy for prostate cancer

**DOI:** 10.1186/s13014-014-0275-6

**Published:** 2014-12-06

**Authors:** Nina-Sophie Hegemann, Matthias Guckenberger, Claus Belka, Ute Ganswindt, Farkhad Manapov, Minglun Li

**Affiliations:** Department of Radiation Oncology, University Hospital Munich, Campus Grosshadern, Marchioninistr. 15, D-81377 Munich, Germany; Department of Radiation Oncology, University Hospital Zurich, Zurich, Switzerland

## Abstract

In the last few years, hypofractionated external beam radiotherapy has gained increasing popularity for prostate cancer treatment, since sufficient evidence exists that prostate cancer has a low α/β ratio, lower than the one of the surrounding organs at risk and thus there is a potential therapeutic benefit of using larger fractionated single doses. Apart from the therapeutic rationale there are advantages such as saving treatment time and medical resources and thereby improving patient’s convenience. While older trials showed unsatisfactory results in both standard and hypofractionated arm due to insufficient radiation doses and non-standard contouring of target volumes, contemporary randomized studies have reported on encouraging results of tumor control mostly without an increase of relevant side effects, especially late toxicity. Aim of this review is to give a detailed analysis of relevant, recently published clinical trials with special focus on rationale for hypofractionation and different therapy settings.

## Rationale for hypofractionation in prostate cancer treatment

The Linear Quadratic Model with its alpha/beta value describes the curvature of cell killing both for tumor control and normal tissue complications in relationship to radiotherapy dose. The alpha/beta ratio is the dose where the linear as well as the quadratic component cause the same amount of cell killing. Generally speaking, the higher the alpha/beta ratio is, the more linear the cell survival curve is. Whereas the lower the alpha/beta ratio is (high beta relative to alpha), the more curved the cell survival curve is. This is important, as tissues with a low alpha/beta are relatively resistant to low doses in contrast to tissues with a high alpha/beta. Thus early responding tissues or rapidly proliferating tumors have a high alpha/beta ratio of more than 10 Gy and late responding tissues or slowly proliferating tumors have a low alpha beta ratio of around 3–5 Gy. Most tumors have a high alpha/beta ratio and can therefore be reasonably treated with conventionally fractionated radiotherapy (using single doses of 1.8 – 2.0 Gy). But some tumors, i.e. melanoma, sarcoma and prostate cancer have a very low alpha/beta ratio and therefore higher single doses can be applied with the aim of achieving better tumor control with approximately the same side effects [1,2]. Furthermore the rationale of fractionation in radiotherapy is also based upon the higher repair-capacity of normal tissue compared to tumor cells, allowing an immediate repair of most radiation-induced sub-lethal lesions in normal tissues between the fractions and thus allowing a relative tumor-specific therapeutic effect [3]. A further reason for hypofractionated prostate cancer treatment is that the surrounding late-responding organs at risk, i.e. rectum or bladder have an estimated α/β-level of 3–5 Gy. Hypothesizing on an α/β-level of 1.5 Gy for prostate cancer and of 3 Gy for rectum, it can be concluded that prostate cancer cells are more responsive to a larger fraction size and that due to a lower α/β-level of prostate cancer cells in comparison with the surrounding late responding tissues there is even a therapeutic gain by using larger fractions sizes [4]. Historically already 15 years ago, there have been attempts to estimate α/β-values for prostate cancer by taking into account results from external beam radiotherapy and brachytherapy. On account of these findings one came up with an α/β-value as low as 1.5 Gy for prostate cancer [5,6]. Fowler et al. have also studied the clinical outcome of patients treated with EBRT or with brachytherapy using I-125 or Pd-103 implants and have also calculated an α/β-value of lower than 2 Gy for prostate cancer [7]. However this must be viewed with caution, since the biological anti-tumor-activity and the dose distributions of high and low dose brachytherapy are not the same as that of EBRT and data sets from different institutions were compared [8]. For example it is still not known, whether there are different fractionation sensitivities for different stages of disease.

To further clarify these issues, Miralbell et al. [2] have recently published data of nearly 6000 patients with different prostate cancer risk groups, all treated with external beam radiotherapy either with standard fractionation (1.8 - 2.0 Gy per fraction; 40% of the patients) or hypofractionation (2.5 - 6.7 Gy per fraction; 60% of the patients). At 5 years tumor control was calculated by using the Phoenix criteria of biochemical relapse free survival, that represents local as well as distant failure and an α/β-value of 1.4 Gy (95% CI = 0.9-2.2) was obtained using the linear-quadratic model [2]. This is so far the most precise estimation of α/β-ratio for EBRT with the largest patient collection. Interestingly, androgen deprivation did not affect the α/β-value, though there was an improvement of bNED for all three risk groups by 5%. Moreover there was not a significant difference in the α/β-values of the three prostate cancer risk-groups [2]. In a further recent analysis of 274 patients with localized prostate cancer hypofractionated radiotherapy of 20 fractions using 3.0 or 3.15 Gy was compared to conventionally fractionated radiotherapy with a median treatment time of 55 days (range 49–66). The calculated α/β-ratio for prostate cancer was 1.86 Gy, with a huge 95%-confidence-interval of 0.7 - 5.1 Gy due to the relatively small number of treated patients [9]. In order to safely apply high single doses, it is vital to know, whether the surrounding organs at risk have a low α/β-ratio as well. A prospective phase II study was conducted by Marzi et al. to estimate the α/β-ratio of the rectum as the main dose-limiting organ at-risk: Patients were either treated with a conventional (80 Gy in 40 fractions over 8 weeks) or a hypofractionated (62 Gy in 20 fractions over 5 weeks) schema. An α/β-ratio of very close to 3 Gy was found for late rectal toxicity and there was no difference of late rectal toxicity between conventional or hypofractionated radiation [10].

### Hypofractionated radiotherapy as primary therapy for prostate cancer

The first studies about hypofractionation were published in the 1990s in the UK, Australia and Canada, where due to long driving distances to the next radiation-oncology department and due to for these countries’ characteristic health care reimbursements the interest in hypofractionation is evident: Lukka et al. conducted in Canada a phase III study comparing conventionally fractionated radiotherapy (66 Gy in 33 fractions) with hypofractionated (52,5 Gy in 20 fractions). Primary outcome was biochemical or clinical failure (BCF), whereas secondary outcomes included presence of tumor at 2 years, survival and toxicity. At 5 year’s follow-up primary outcome (52,95% vs. 59,95%), as well as acute toxicity were both worse in the hypofractionated in comparison to the conventionally fractionated arm, although without a difference in late toxicity, in 2-year post-radiotherapy biopsy or overall survival [11]. In Australia Yeoh et al. conducted a study from 1996 until 2003 comparing toxicity and efficacy of radiation therapy for localized carcinoma of the prostate using a hypofractionated (55 Gy/20 fractions/4 weeks) vs. a conventionally fractionated (64 Gy/32 fractions/6.5 weeks) dose schedule. At a follow-up for a median of 90 months biochemical relapse free survival according the Phoenix criteria was significantly better in the hypofractionated group than in the conventionally fractionated group, but not overall survival. Gastrointestinal and genitourinary toxicity did not differ between the two dose schedules. According to Yeoh et al. there is a great therapeutic advantage of hypofractionated compared to conventionally fractionated dose schedule for radiotherapy of prostate cancer [12,13]. Both studies need to be critically commented on: First in both studies overall doses were underdosed compared to nowadays conventionally fractionated overall doses of 74.0-80.0 Gy. Second planning was undertaken mostly by 2D-plans, and not at least by nowadays standardly used 3D-plans or even IMRT plans. Therefore these two studies resulted in a much higher toxicity rate than one would expect nowadays. Third these trials did not consider the α/β-ratio of prostate cancer before starting and comparing hypofractionation to a conventionally fractionated schedule. Therefore these two studies can be seen as a first experiment with hypofractionation and have shown, that it is feasible, but they lack in reasonable results due to older planning methods and underdose. Modern studies are based on an α/β-ratio of prostate cancer of 1.5 Gy and were conducted to either show the equivalent effectiveness between the two fractionation schedules with reduced side effects in the hypofractionated arm or to show a higher effectiveness with equal toxicity due to a higher biological effective dose.

The results of several such prospective trials with adequate dose have been published now (Table 1). Arcangeli et al. conducted a study in high-risk prostate cancer patients comparing conventional fractionation (80 Gy/2 Gy/8 weeks) to hypofractionation (62 Gy/3.1 Gy/5 weeks). At a follow-up of a median of 70 months there was a significant risk reduction in biochemical failure (10.3 %), but not in local or distant failure, when considering the entire group of patients. Whereas there was a significant increase of 5-year freedom from biochemical, local and even distant failure for a subgroup of patients with a pretreatment PSA level of 20 ng/ml or less. This might be due to a greater local effectiveness of hypofractionation on a smaller tumor burden and thus due to the achievement of local tumor control before the occurrence of metastatic spread. Arcangeli et al. have therefore shown that hypofractionation in high risk prostate cancer is at least isoeffective in regard of biochemical control [14]. Dearnaley et al. presented a preliminary safety analysis on side effects of their CHHiP trial (Conventional or Hypofractionated High-dose Intensity Modulated Radiotherapy in Prostate Cancer). Patients were either treated with conventionally fractionated (74 Gy/2 Gy) or hypofractionated (60 Gy/3 Gy or 57 Gy/3 Gy) intensity modulated radiotherapy and all patients received 3–6 months of neoadjuvant androgen suppression. The primary endpoint was the proportion of patients with grade 2 or worse toxicity at 2 years on the RTOG scale. Between the three groups there was no significant difference in regard of bowel toxicity ≥ grade 2 or bladder toxicity ≥ grade 2 at a median follow-up of 50.5 months and the overall number of incidents was low. The effectiveness of these three radiotherapy schemes has not yet been published [15]. Another two fairly recently published studies have been performed with modern radiation tools like IMRT [16,17]. Pollack et al. treated 303 patients with favorable- to high-risk prostate cancer with either conventionally fractionated IMRT (CIMRT) with 76 Gy in 38 fractions at 2.0 Gy per fraction or with hypofractionated IMRT (HIMRT) with 70.2 Gy in 26 fractions at 2.7 Gy per fraction (equivalent to 84.4 Gy in 2.0 Gy fractions). With a median follow up 68.4 months there was no significant difference in regard of biochemical and/or clinical disease failure (BCDF) (21.4% for CIMRT vs. 23.3% for HIMRT) nor in regard of late toxicity, although patients with compromised urinary function before enrollment had significantly worse urinary function after HIMRT. Although this study did not result in a significant reduction of BCDF, patients had the advantage of 2.5 fewer treatment weeks and it was shown, that hypofractionated IMRT could safely be applied apart from patients with compromised urinary function.

**Table 1:** **Hypofractionated primary radiotherapy for prostate cancer**

**Institution**	**Number of patients**	**Fractionation (total dose/singel dose/fractions)**	**EQD for tumor α/β-ratio 1.4Gy**	**EQD for normal tissue α/β-ratio 3Gy**	**Follow-up**	**Acute GU toxicitiy**	**Late GU toxicity**	**Acute GI toxicity**	**Late GI toxicity**	**Therapeutic outcomes**
Rome, Italy [14]	168 pat.	Arm I: 80Gy/2Gy/40 fractions; ArmII: 62Gy/3.1Gy/20 fractions, 4x/week.	82.1Gy	74.2Gy	70 months	Arm I: 40% ≥ II° GI, Arm II: 47% ≥ II° GI.	Arm I: 16% ≥ II° GI, Arm II: 11% ≥ II° GI.	Arm I: 21% = II° GI, Arm II: 35% = II° GI.	Arm I: 17% ≥ II° GI, Arm II: 14% ≥ II° GI.	Hypofraction-RT is not inferior to conventional RT, potentially even better for high-risk pat. (iPSA > 20 ng/ml, GS > 7, cT > 2c).
11 UK centres [15]	Arm I: 153 pat. 74Gy; Arm II 153 pat. 60Gy and 151 pat. 57Gy.	Arm I: 74Gy/2Gy/37 fractions; Arm II: 57-60Gy/3Gy/19-20 fractions	73.8/77.6Gy	68.4/72Gy	50.5 months	-	3 pat. (2 · 2%) in 74Gy group, 3 (2 · 2%) in 60Gy group, and 0 in 57Gy group ≥ II° GU.	-	6 pat. (4 · 3%) in Arm I ≥ II° GI RTOG, 5 pat. (3 · 6%) in Arm II, 2 (1 · 4%) in 57Gy group.	-
Fox Chase, Philadelphia [16]	307 pat. (ASTRO Update 2011)	Arm I: 76Gy/2Gy/28 fractions; Arm II: 70.2Gy/2.7Gy/26 fractions	84.7Gy	80Gy	5 years	Arm I: 54% > II°; 2% > III°; Arm II: 40% > II°; 8% > III°.	Arm I: 8.3%; Arm II: 18.3% at 5 years.	Arm I: 8% > II° GI; Arm II: 18% > II° GI.	-	biochemical recurrence 21.5% vs. 21.9% at 5 years
MDACC [17]	101 pat. in CIMRT, 102 pat. in HIMRT arm. For all pat. 28% low-risk, 71% intermed.-risk, 1% high-risk	CIMRT arm: 75.6 Gy/1.8Gy/42 fractions; HIMRT arm: 72Gy/2.4Gy/30 fractions	85,5Gy	81Gy	6 years	-	At 5 years, CIMRT: 15% I°, 14% II°, 1% III°; HIMRT: 10% I°, 15% II°, 0% III°.	-	At 5 years, CIMRT: 17% I°, 4% II°, 1% III°;HIMRT: 26% I°, 9% II°, 2% III°.	-
Ontario, Canada [11]	Arm I: 470, Arm II: 436 pat.	Arm I: 66Gy/2Gy/33 fractions; Arm II: 52.5Gy/2.63Gy/20 fractions	62.2Gy	59.1Gy	5.7 years	Arm I: 7% ≥ III° GU, Arm II: 11.4% ≥ III° GU.	Arm I: 1.9% ≥ III° GU, Arm II: 1.9% ≥ III° GU.	Arm I: 2.6% ≥ III° GI, Arm II: 4.1% ≥ III° GI.	Arm I: 1.3% ≥ III° GI, Arm II: 1.3% ≥ III° GI.	at 5 years, BCF in Arm I 53%, in Arm II 60%.
Adelaide, Australia [13]	Arm I: 108 pat.; Arm II: 109 pat.	Arm I: 64Gy/2Gy/32 fractions; Arm II: 55Gy/2.75Gy/20 fractions	67.1Gy	63.25Gy	90 months	-	no signif. diff. between 2 groups at 5 years	-	no signif. diff. between 2 groups	biochemical relapse-free survival at 90 months 53% in hypofraction Arm vs. 34% in control Arm.
Vilnius, Lithuania [50]	91 pat. low- and intermed.-risk	Arm I: 74Gy/2Gy/37 fractions; Arm II: 57Gy = 13×3Gy + 4×4.5Gy	84.9Gy	73.8Gy	3 months	Arm I: 21 (47.7%) and Arm II: 9 (19.1%) = II° GU.	-	Arm I: 10 (22.7%) and Arm II: 8 (17%) = II° GI.	-	-
Milan, Italy [51]	337 all cT1-2. 40.9% low-risk; 43.3% intermed-risk; 14.2% high-risk.	70.2Gy/2.7Gy/26 frations	84.7Gy	80Gy	19 months	35% ≥ II° GU, 6.2% ≥ III° GU.	10.4% ≥ II° GU, 1.6% ≥ III° GU.	11.3% ≥ II° GI, 1.2% ≥ III° GI.	7.5% ≥ II° GI, 1.3% ≥ III° GI.	-
Cleveland Ohio [18]	770 pat, 34% low-risk, 28% intermed.-risk, 38% high-risk .	70Gy/2.5Gy/28 fractions, but mean target dose was 75.3Gy at 2.7Gy.	80.3Gy, 90.8Gy (mean target dose)	77Gy	45 months	48% I°, 18% II°, 1% III° RTOG GU.	4.3% I°, 5.1% II°, 0.1%(1 pat.) III° RTOG GU.	40% I°, 9% II° RTOG GI.	5.9% I°, 3.1% II°, 1.3% III°, 0.1%(1 pat.) IV° RTOG GI.	nadia + 2 ng/ml bRFS at 5 years 83%, and 94%, 83%, 72%.

Besides the prospective studies mentioned above, Kupelian et al. reported on their experience of 770 prostate cancer patients treated with hypofractionated radiotherapy (70.0 Gy/2.5 Gy) in a retrospective analysis. Both tumor control and toxicity rate were acceptable and comparable with data of conventionally fractionated radiotherapy studies [18].

Several phase III non-inferiority trials are going on, such as the RTOG 0415 and PROFIT trials. The RTOG 0415 trial compares a 70.0 Gy at 2.5 Gy-schedule with a 73.8 Gy at 1.8 Gy-schedule. This trial is primarily testing non-inferiority, and secondarily toxicity. If the α/β-ratio of prostate cancer is around 10, this trial will be able to demonstrate at least iso-effectiveness between the two fractionation schemes. However if the α/β-ratio is closer to 1.5 Gy, as it is widely assumed to be, the hypofractionated schema should result in a higher tumor control. This trial is closed to accrual, but is not yet published. So far it can be stated that hypofractionated schemes are at least as effective and tolerable as conventionally fractionated schemes. Whether hypofractionation causes less side effects at a higher tumor control still needs to be awaited and to be investigated in further trials.

### Adjuvant or salvage hypofractionated radiotherapy

The significance of adjuvant radiotherapy has been clearly proven in three phase III randomized trials [19-22]. Adjuvant radiotherapy with a dose range from 60–64 Gy after radical prostatectomy should be deployed in patients with unfavorable risk factors such as pT3-prostate cancer and/or positive surgical margins, especially in presence of high Gleason scores [19-22]. If a biochemical failure occurs, a salvage radiotherapy should be done. As most trials on adjuvant or salvage radiotherapy were conducted with a conventionally fractionated scheme, evidence on hypofractionation for these indications is scarce and only few trials on this topic exist.

There are two Italian studies on adjuvant radiotherapy with a hypofractionated scheme [23,24]; Table 2). Ippolito et al. performed an IMRT based dose-finding trial with four increasing simultaneous integrated boosts (56.8 Gy/2.27 Gy; 59.7 Gy/2.39 Gy; 61.25 Gy/2.45 Gy and 62.5 Gy/2.5 Gy) to the prostate bed while irradiating the pelvic lymph nodes (45 Gy/1.8 Gy). No dose-limiting toxicity was seen at a median follow-up time of 19 months and therefore the recommended dose was 62.5 Gy in 2.5 Gy/fraction [24]. In 2008 Cozzarini et al. also reported on hypofractionated adjuvant radiotherapy of 50 patients, using helical tomotherapy. They applied 58 Gy in 20 fractions and assessed toxicity in comparison to their institutional 3D-CRT with a conventionally fractionated dose scheme. Initially, they observed no difference in acute grade 2–3 genitourinary and acute grade 2 gastrointestinal toxicity [23]. However, in the update report now, they presented an unexpected high rate of severe late urinary toxicity (16.5% patients at grade 3–4) in 247 patients after a median follow-up of 68 months [25]. More importantly, the fraction size has been shown to be an independent prognostic factor for severe late urinary toxicity in the univariable and multivariable Cox analysis. Thus the authors appealed special caution of using hypofractionated schedule in a postprostatectomy setting due to the risk of severe late urinary toxicity.

**Table 2:** **Hypofractionated adjuvant/salvage radiotherapy**

**Reference**	**Study design**	**Institution**	**Patient collection**	**Fractionation(total dose/singel dose/fractions)**	**EQD for tumor α/β ratio 1.4Gy**	**EQD for normal tissue α/β ratio 3Gy**	**IMRT**	**Follow-up**	**Acute GU toxicitiy**	**Late GU toxicity**	**Acute GI toxicity**	**Late GI toxicity**	**Therapeutic outcomes**
Cozzarini, C. [23]	Prospective phase I-II for adjuvant RT	Milan, Italy	247 patients	65.8Gy/2.35Gy/28 fractions adj. RT for 117 pat.; 71.4-72.8Gy/2.55Gy/28 fractions salvage RT for 80 pat.; 58Gy/2.9Gy/20 fractions for 50 pat. Conventional arm 929 pat. 70.2Gy/1.8Gy/39 fractions	72.6Gy adjuvant RT; 83.0Gy salvage RT; 73.4Gy for the other 50 pat.	α/β ratio = **5Gy** for late GU toxicity! 69.14Gy adjuvant RT; 77.1Gy salvage RT; 65.5Gy for the other 50 pat.	Tomo-RT	68 months median	-	41/247 (16.5%) ≥ III° GU in hypofraction arm; 72/929 (7.7%) in conventional arm	-	-	-
Kruse, T.J. [26]	Retrospective for salvage RT	Madison, Wisconsin	108 patients	65Gy/2.5Gy/26 fractions	74.6Gy	71.5Gy	IMRT	32.4 months median	8 pat. (7%) II° and 1 pat. III° GU RTOG.	16 pat. (15%) II° GU RTOG.	15 pat. (14%) II GI RTOG.	4 (4%) pat. II° GI RTOG.	freedom from biochem. failure at 4 years 67% ± 5.3%.
Ippolito, E. [24]	Prospective phase I for dose-escalation, adjuvant RT	Campobasso, Italy	25 patients	7 pat. 56.8Gy/2.27Gy/25 fractions; 6 pat. 59.7Gy/2.39Gy/25fractions; 6 pat. 61.25Gy/2.45Gy/25fractions; 6 pat. 62.5Gy/2.5Gy/25 fractions	7 pat. 61.3Gy; 6 pat. 66.5Gy; 6 pat. 69.4Gy; 6 pat. 71.7Gy.	7 pat. 59.9Gy; 6 pat. 64.4Gy; 6 pat. 66.8Gy; 6 pat. 68.8Gy.	IMRT	19 months median	9/25 (36%) II° GU.	-	5/25 (20%) II° GI.	-	-
Lee, W. [52]	Retrospective for salvage RT	Manchester	37 patients	50-52.5Gy/2.5-2.63Gy/20 fractions	57.4-62.2Gy	55-59.1Gy	-	30.6 months median	0% II° GU.	16 pat. I° GU, 0 pat. II° GU.	0% II° GI.	4 pat. I° GI, 1 pat. II° GI.	3-year disease-free survival is 74%.

In the setting of a biochemical failure, Kruser et al. published their experience of hypofractionated radiotherapy (65.0 Gy/2.5 Gy in 26 fractions) on 108 patients with a median PSA elevation of 0.44 ng/ml after prostatectomy. The therapeutic results were encouraging: There was a 67% ± 5.3% freedom from biochemical failure at 4 years and there was only moderate toxicity with only one acute grade 3 genitourinary and with no acute grade 3 gastrointestinal toxicity and no late grade 3 toxicities [26]. Hypofractionated salvage radiotherapy in this study thus resulted in a low rate of acute and late toxicity with good tumor control while reducing overall treatment time.

As there is apart from these three studies only scarce information on this topic, Krause et al. have initiated the PRIAMOS trial to investigate safety and feasibility of hypofractionated treatment of the prostate bed alone as well as of the pelvic lymph nodes [27]. A total of 80 prostate cancer patients shall be enrolled, of whom 40 patients with a low risk of lymph node involvement will receive 54 Gy/3 Gy in 18 fractions to prostate bed only, whereas 40 patients with a high risk for lymph node involvement will receive 45 Gy/2.5 Gy in 18 fractions to the lymph nodes additionally. Assuming an α/β-ratio of 1.5 Gy for prostate cancer the biological effective dose (BED) would be 69.4 Gy for the prostate bed and 51.4 Gy for the pelvic lymph nodes and therefore all well below the tolerance doses of the respective surrounding organs at risk, such as small bowel (BED 47.5 Gy; α/β-ratio 7 Gy) and rectum (BED 63 Gy, α/β-ratio 4 Gy). Patient accrual started in March 2012 and end of accrual is planned for March 2014. Therefore safety and tumor control of prostate bed and lymph nodes both treated with a hypofractionated schema still needs to be awaited. All in all there is a hint so far for a potential advantage of hypofractionated radiotherapy in adjuvant and salvage treatment for prostate cancer, especially in the context of resource-saving and patients’ convenience. However, on the other side, the hypofractionation-associated late toxicity may be also significantly increased so that now in the absence of solid evidence careful attention should be taken proposing hypofractionated radiotherapy in the adjuvant or salvage setting outside well designed prospective clinical trials.

### Hypofractionated radiotherapy with pelvic lymph node irradiation

Initially, hypofractionated radiotherapy was performed to solely irradiate the prostate. With the wider spread of 3D-conformal radiation therapy technique, as well as of IMRT, a more sophisticated dose delivery to the prostate/prostate bed with a simultaneous radiation of pelvic lymph nodes has been integrated in recent trials for patients with high risk for lymph node metastases. Since radiotherapy-induced toxicities do not only depend on the radiation dose but also on the irradiated volume of organs at-risk [28], the addition of pelvic lymph nodes’ irradiation might increase the dose exposure, i.e. of rectum or bladder, and thus the side effects.

Until now, several prospective and retrospective trials have been published [24,29-34] on hypofractionated treatment of the prostate/prostate bed with simultaneous conventionally fractionated irradiation of the pelvic lymph nodes (Table 3). So far apart from the above mentioned and currently accruing trial on hypofractionated irradiation of pelvic lymph nodes, no study has been published so far on hypofractionation in pelvic lymph nodes’ treatment. McDonald et al. [31] retrospectively collected data on toxicity of patients, that either received an IMRT based prostate-only radiotherapy (PORT, 70.0 Gy/2.5 Gy) or an additional, conventionally fractionated whole-pelvic radiotherapy (WPRT, 50.4 Gy/1.8 Gy). They hypothesized that the treatment of pelvic lymph nodes will result in an increased toxicity compared to hypofractionated treatment of prostate only. They therefore retrospectively performed a daily dosimetry for patients, who developed grade 2 or higher late toxicities in order to correlate the dose-levels to the rectum with the occurred toxicity. Concerning acute gastrointestinal (GI) and genitourinary (GU) toxicity there was no statistically significant difference with the addition of elective node irradiation (ENI). Whereas in regard to late grade ≥2 rectal toxicity there was a statistically significant difference between WPRT-group (18%) and PORT-group (0%) at a median follow-up of 41.1 months. When retrospectively contouring the daily CT-scans of patients, who developed a grade ≥ 2 rectal toxicity, the combined average daily deviation of the actual rectal volume from the planned volume was 12.7% and 88% of all fractions delivered a higher V70 than originally planned. This study therefore showed that the addition of ENI leads to a significant higher rate of late grade ≥2 rectal toxicity and confirms the above mentioned hypothesis that the dose exposure of the rectum increases with the irradiation of larger volumes, like the inclusion of pelvic lymph nodes. This finding is consistent with results of studies with conventionally irradiated prostate and pelvic lymph nodes, like GETUG-01 study [35] or with results of hypofractionated irradiation of prostate with conventional irradiated pelvic lymph nodes, like the study of McCammon et al. [30], in which the actuarial rate of late grade ≥2 GI events was 13.4% at 6 years. Furthermore there is the study of Adkison et al. on dose-escalated treatment of the pelvic lymph nodes with 56 Gy in conventional fractionation combined with hypofractionated treatment of the prostate (70 Gy in 2.5 Gy fractions), that was shown to be well tolerated [29]. Recently Guckenberger et al. presented outcome and toxicity of 150 prostate cancer patients that either received an IMRT with mean total doses of 73.9 Gy in 32 fractions or with mean total doses of 76.2 Gy in 33 fractions [34]. The pelvic lymph nodes were treated in 41 high-risk patients with an overall dose of 45.0 Gy and a single fraction dose of 1.8 Gy. At a median follow-up of 50 months only two patients suffered from late grade 3 GI toxicity and > 80% of patients were free from any GI toxicity during follow-up. Acute GU toxicity grade 1–2 was observed in 85% of the patients with most patients recovering from it within 6 weeks after treatment. Interestingly the proportion of patients with GU toxicity grade ≥2 was <10% at 6–12 months after radiotherapy but increased continuously to 22.4% at 60 months. On univariate analysis it was shown that no clinical or treatment factor influenced significantly the risk of acute GU toxicity ≥2 and that treatment of the whole pelvis had no effect on the rates of late GU toxicity grade ≥2. With a 5-year freedom from biochemical failure rate (FFBF) of 82% for all patients and an especially favorable rate of 78% for high-risk patients Guckenberger et al. showed that a moderately hypofractionated IMRT also with a simultaneous irradiation of pelvic lymph nodes can be safely performed. So far hypofractionated treatment of prostate in combination with conventionally fractionated irradiation of pelvic lymph nodes seems to be feasible at an acceptable toxicity rate, but data of the study of Krause et al. [27] on hypofractionated treatment of prostate as well as of pelvic lymph nodes are most eagerly awaited.

**Table 3:** **Hypofractionated radiotherapy including pelvic nodes**

**Reference**	**Study design**	**Number of patients**	**Fractionation (total dose/singel dose/fractions)**	**pelvic RT dose schema**	**EQD for tumor α/β-ratio 1.4Gy**	**EQD for normal tissue α/β-ratio 3Gy**	**Follow-up**	**Acute GU toxicitiy**	**Late GU toxicity**	**Acute GI toxicity**	**Late GI toxicity**
McDonald, A.M. [31]	Retrospective	57 PORT and 31 WPRT	70Gy/2.5Gy/28 fractions	50.4Gy/1.8Gy/28 fractions	80.3Gy	77Gy	41 months	18/31(58%) in PORT, 28/57(49%) in WPRT ≥2°	4/57(7%)in WPRT, 0% in PORT ≥ III°	7/31(23%) in PORT, 23/57 (40%) in WPRT ≥ II°	0% in PORT, 10/57(18%) in WPRT ≥ II°
McCammon, R. [30]	Retrospective	30	70Gy/2.5Gy/28 fractions	50.4Gy/1.8Gy/28 fractions	80.3Gy	77Gy	24 months	36.7% ≥2°	10% ≥ II°	20%	13% ≥ II°
Adkinson, J.B. [29]	Phase I prospective	53	70Gy/2.5Gy/28 fractions	56Gy/2Gy/28 fractions	80.3Gy	77Gy	25.4 months	20/53(38%) ≥2°	14/53(27%) ≥ II°	17/53(32%) ≥ II°	4/53(8%) ≥ II°
Pervez, N. [32]	Phase II prospective	60 high-risk	68Gy/2.72Gy/25 fractions	45Gy/1.8Gy/25 fractions	82.4Gy	77.8Gy	3 months	34(40%) ≥ II°	-	21(35%) ≥ II°	-
Quon, H. [33]	Prospective phase I-II	97 pat. High-risk	67.5Gy/2.7Gy/25 fractions	45Gy/1.8Gy/25 fractions	81.4Gy	77Gy	39 months median	50% I°, 39% II°, 4% III°	9% I°, 5% II°, 3% III°, 1% IV°.	4% pat. 0°, 59% I°, 37% II°	54% pat. 0°, 40% I°, 7% II°
Guckenberg, M. [34]	150 consecutive patients	109 PORT and 41 WPRT	73,9Gy/2,31Gy/32 fx; 76.2Gy/2.31Gy/33 fx.	45Gy/1.8Gy/25 fractions	80.6Gy;83.1Gy.	78.5Gy; 80.9Gy.	50 months median	85% pat. I°-II°	22.4% Pat. ≥ II° at 60 months; less than 5% pat. III°.	-	2 pat. ≥III°
Fonteyne, V. [53]	Prospective phase I	31 patients	69.3/2.77Gy/25 fractions	50Gy/2.0Gy/25 fractions	85Gy	80Gy	3 months median	14/31 (45%) II°, 3/31 (9.7%) III°	-	14/31 (45%) II° lower GI toxicity	-
Zilli, T. [54]	Prospective trial	78 pat.	50.4Gy/1.8Gy/28 fractions +6x4Gy boost (twice weekly)	50.4Gy/1.8Gy/28 fractions	85.2Gy with 1.5Gy alpha/beta	-	57 months	~1% = III°	5 year suivival rate without II° GU toxicity 79.1 ± 4.8%	~1% = III°	5 year suivival rate without II° GI toxicity 84.1 ± 4.5%

### Role of IMRT/IGRT in hypofractionation

The use of improved technology has fostered the increasing interest in hypofractionated radiotherapy for prostate cancer. Multiple studies have confirmed the importance of delivering sufficiently high doses to prostate in order to cure patients. Due to inverse planning, the key factor of intensity modulated radiotherapy (IMRT) dose distributions with a maximum sparing of organs at risk are possible. Furthermore with the advances in imaging and onboard verification systems as part of image guided radiotherapy (IGRT) the capabilities of IMRT enable an even more sophisticated dose distribution and are the reasons for further dose escalation and hypofractionated schemes [36]. IMRT and IGRT are the tools that allow the radiooncologist to safely deliver escalated single and/or overall doses and are the primary intellectual prerequisite to allow these therapeutic advances. While, as stated above, hypofractionated radiotherapy may probably result a higher biological equivalent dose to prostate cancer than to the surrounding normal tissues due to the lower alpha/beta value of prostate cancer and thus protect organs at risk in the biological perspective, IMRT and IGRT with the geometric advantages of dose delivery may potentially further increase the therapeutic ratio of hypofractionated radiotherapy. Here we listed the relevant hypofractionated trials using IMRT and IGRT in Table 4.

**Table 4:** **Hypofractionated IMRT/IGRT trials**

**Reference**	**Study design**	**Number of patients**	**Fractionation (total dose/ singel dose/fractions)**	**EQD for tumor α/β-ratio 1.4Gy**	**EQD for normal tussues α/β-ratio 3Gy**	**IGRT**	**Follow-up**	**Acute GU toxicitiy**	**Late GU toxicity**	**Acute GI toxicity**	**Late GI toxicity**	**Therapeutic outcomes**
Arcangeli, G. [14]	Phase III prospective	168 pat.	Arm I: 80Gy/2Gy/40 fractions, ArmII: 62Gy/3.1Gy/20 fractions, 4x/week.	82.1Gy	74.2Gy	daily portal imaging	70 months	-	Arm I: 16% ≥ II° GI, Arm II: 11% ≥ II° GI., at 3 years.	-	Arm I: 17% ≥ II° GI, Arm II: 14% ≥ II° GI, at 3 years.	Hypofraction-RT is not inferior to conventional RT.
Dearnaley, D. [15]	Phase III prospective	Arm I: 153 pat. 74Gy; Arm II 153 pat. 60Gy and 151 pat. 57Gy.	Arm I: 74Gy/2Gy/37 fx; Arm II: 57-60Gy/3Gy/19-20 fx.	Arm II: 73.8/77.6Gy	Arm II: 68.4/72Gy	no	50.5 months	-	At 2 years, 3 pat. (2 · 2%) in 74Gy group, 3 (2 · 2%) in 60Gy group, and 0 in 57Gy group ≥ II° GU.	-	At 2 years, 6 pat. (4 · 3%) in Arm I ≥ II° RTOG, 5 pat. (3 · 6%) in 60Gy group, 2 (1 · 4%) in 57Gy group.	-
Pollack, A. [16]	Phase III prospective	307 (ASTRO Update 2011)	Arm I: 76Gy/2Gy/28 fx; Arm II: 70.2Gy/2.7Gy/26 fx.	Arm II: 84.7Gy	Arm II: 80Gy.	no	5 years	Arm I: 54% > II°; 2% > III°; Arm II: 40% > II°; 8% > III°.	Arm I: 8.3%; Arm II: 18.3% at 5 years	Arm I: 8% ≥ II°; Arm II: 18% ≥ II°	4.5% ≥ II° GI.	biochem. recurrence 21.5% vs. 21.9% at 5 years
McDonald, A.M. [31]	Retrospective	57 PORT and 31 WPRT	70Gy/2.5Gy/ 28 fractions	80.3Gy	77Gy	CBCT daily	41 months	18/31(58%) in PORT, 28/57(49%) in WPRT ≥ II°	4/57(7%)in WPRT, 0% in PORT ≥ III° GU	7/31(23%) in PORT, 23/57 (40%) in WPRT ≥ II° GI	0% in PORT, 10/57(18%) in WPRT ≥ II° GI	-
Adkinson, J.B. [29]	Phase I prospective	53 pat.	70Gy/2.5Gy/28 fractions	80.3Gy	77Gy	yes	25.4 months	20/53(38%) ≥ II° GU	14/53(27%) ≥ II° GU	17/53(32%) ≥ II° GI	4/53(8%) ≥ II° GI	biochem. control (nadir +2) 81.2 ± 6.6%.
Jereczek-Fossa, B.A. [51]	Prospective longitudinal follow-up	337 pat. cT1-2, 40.9% low-risk; 43.3% intermed-risk; 14.2% high-risk.	70.2Gy/2.7Gy/26 frations	84.7Gy	80Gy	BAT 72%, stereo X-ray 16.4%, CBCT 11.9% pat.	19 months	35% ≥ II° GU, 6.2% ≥ III° GU.	10.4% ≥ II° GU, 1.6% ≥ III° GU.	11.3% ≥ II° GI, 1.2% ≥ III° GI.	7.5% ≥ II° GI, 1.3% ≥ III° GI.	-
Kupelian, P. A. [18]	Retrospective	770 pat, 34% low-risk, 28% intermed.-risk, 38% high-risk D'Amico criterien.	70Gy/2.5Gy/28 fractions, but mean target dose was 75.3Gy at 2.7Gy!	80.3Gy with 1.4Gy α/β-ratio (83.8Gy with 3.5Gy α/β-ratio in publication)	77Gy	IGRT with BAT tranabdominal ultrasound	45 months	33% Pat. 0°, 48% I°, 18% II°, 1% III° RTOG	90.5% pat. 0°, 4.3% I°, 5.1% II°, 0.1%(1 pat.) III° RTOG	51% pat. 0°, 40% I°, 9% II° RTOG	89.6% pat. 0°, 5.9% I°, 3.1% II°, 1.4% ≥ III° RTOG	At 5 years 94%, 83%, 72% for low-/intermed./high-risk respectively (Nadir + 2 ng/ml)
Quon, H. [33]	Prospective phase I-II	97 pat. High-risk	67.5Gy/2.7Gy/25 fractions	81.4Gy	77Gy	IGRT with gold marker	39 months median	8% pat. 0°, 50% I°, 39% II°, 4% III° **CTCAE**	82% pat. 0°, 9% I°, 5% II°, 3% III°, 1% IV°.	4% pat. 0°, 59% I°, 37% II°	54% pat. 0°, 40% I°, and 7% II°	4 year bFFS 90.5%.
Martin, J. [40]	Prospective phase I-II	92 pat., mainly intermed./low risk	60Gy/3Gy/20 fractions	77.6Gy	72Gy	IGRT with gold marker	38 months median	32% pat. 0°, 43% I°, 25% II° RTOG	90% pat. 0°, 7% I°, 3% II° RTOG	66% pat. 0°, 22% I°, 11% II°, 1% IV° RTOG	93% pat. 0°, 2% I°, 4% II° RTOG	3 year biochemical control 76%.

One of the most important findings on this topic are the already above mentioned preliminary safety results of the trial CHHiP (Conventional or Hypofractionated High-dose Intensity Modulated Radiotherapy in Prostate Cancer), where patients were either treated with conventional (74 Gy/2 Gy) or hypofractionated (60 Gy/3 Gy or 57 Gy/3 Gy) intensity modulated radiotherapy (IMRT) and all were given 3–6 months of neoadjuvant androgen suppression. Between the three groups there was no significant difference in regard of bowel toxicity or bladder toxicity ≥ grade 2 at a median follow-up of 50.5 months and the results from this pre-planned safety analysis of the first 457 patients, that were enrolled in the CHHiP trial, suggest that high dose hypofractionated schedules using single doses of 3 Gy in combination with intensity-modulated radiotherapy are safe [15]. Besides deploying IMRT, delineation of clinical target volumes and defining its safety margins are vital for hypofractionation. Pollack et al. first reported in 2006 on dosimetry and acute toxicity of their randomized hypofractionation dose escalation trial, in which they compared a conventionally fractionated Arm I (76 Gy in 38 fractions) with a hypofractionated Arm II (70.2 Gy in 26 fractions). They applied different planning target volume (PTV) margins in Arm I and II. Margins of PTV were posteriorly 5 mm in Arm I vs. 3 mm in Arm II and in all other dimensions 8 mm in Arm I vs. 7 mm in Arm II. PTV D95% was at least the prescription dose. Therefore the mean PTV doses for Arms I and II were 81.1 Gy and 73.8 Gy. Due to these planning conditions there were no differences in overall acute gastrointestinal (GI) or genitourinary (GU) toxicity, although there was a slight, but significant increase in Arm II GI toxicity during weeks 2, 3 and 4 [37]. In 2013 Pollack et al. presented an update of this study (303 assessable patients) with emphasis on late toxicity and biochemical and/or clinical disease failure (BCDF). At a median follow-up of 68.4 months there were statistically no significant differences in late toxicity between the two arms, however in subgroup analysis, patients with compromised urinary function before enrollment had significantly worse urinary function after hypofractionated radiotherapy and therefore might not be ideal candidates for a hypofractionated approach. In regard of 5-year rates of BCDF the study of Pollack et al. did not show a significant difference (21.4% in conventionally fractionated group versus 23.3% in hypofractionated group) with no difference in the use of androgen deprivation therapy in the two groups [16]. Reducing safety margins of PTV and thereby reducing a possible overlap with the surrounding organs at risk, particularly the rectum is only possible by using image guided radiotherapy (IGRT). Only the use of IGRT allows the control of inter- and intrafraction motions of target volumes and therefore allows a more precise dose delivery with smaller safety margins. This has not only been proven in conventionally fractionated studies with dose escalation, like the study of Pinkawa et al. [38], but also for hypofractionated regimes. Kupelian et al. first published in 2000 about their “Cleveland experience” on short-course intensity-modulated radiotherapy (70.0 Gy in 28 fractions) in the treatment of localized prostate cancer while using daily transabdominal ultrasound for localization [39]. They reported on their first 51 patients that were treated with an intensity modulated radiotherapy plan with safety margins for the planning target volume (PTV) of 4 mm posteriorly, 8 mm laterally and 5 mm in all other directions. The location of the prostate gland was verified daily with the BAT transabdominal ultrasound system and patient position adjustments were performed accordingly. These patients were compared in regard to acute bladder and rectal toxicity with prostate cancer patients, that have been treated with a more standard conformal technique (78 Gy/2 Gy per fraction) and there was no statistical difference in the frequencies of acute bladder or acute rectal toxicity [18]. Furthermore the insertion of fiducial markers (gold seeds) in the prostate helps the radiooncologist to daily monitor deviations regardless of whether they use ultrasound or a CT based system for IGRT [40]. For sure it is possible to do daily low dose CT controls, but especially for the prostate gland IGRT with ultrasound can easily be used. All in all IMRT and IGRT are the requirements to safely apply hypofractionated schemes.

### Stereotactic body radiotherapy and intense hypofractionation

Stereotactic body radiotherapy (SBRT) with a fraction size larger than 5 Gy has emerged as a further option to treat prostate cancer with the potential advantage for the patient of an even shorter treatment time. However, on the other side, it remains controversial how far the linear-quadratic model is still valid using such a large dose size. So far, most clinical trials on stereotactic body radiotherapy were performed for organ-confined low risk prostate cancer [41-46]. One of the pioneers in this field is C.R. King. In 2012 he published results of their ongoing prospective Phase II clinical trial using SBRT for low-risk prostate cancer. From 2003 through 2009, 67 patients with clinically localized low-risk prostate cancer received 36.25 Gy in 5 fractions with CyberKnife system as the delivery technology and without any further therapy, such as hormonal ablation. At a median follow-up of 2.7 years late RTOG Grade III, II and I bladder toxicities were seen in 2, 3 and 13 patients with no late urinary Grade IV toxicity. Late rectal Grade III, II and I toxicities were seen in 0, 1 and 7 patients with no persistent rectal bleeding. The 4-year Kaplan-Meier PSA relapse-free survival was 94% and is similar to other definitive treatments [43]. Even though these are excellent results, C.R. King and his co-publishers advice to act with caution and to enroll patients for SBRT primarily in clinical trials, as so far long term results for therapeutic efficacy and toxicities are not completely sufficient.

He further reported on a prospective Phase II clinical trial that confirmed the advantage of every-other-day treatment. In this trial forty-one low-risk prostate cancer patients with 6 months’ minimum follow-up received as well 36.25 Gy in five fractions of 7.25 Gy on 5 consecutive days or every-other-day with image-guided SBRT using as well the CyberKnife. In this study a reduced rate of severe rectal toxicities was observed with every-other-day vs. 5 consecutive days treatment regimen (0% vs. 38%, p = 0.0035). Given their observations, they favor treating with a longer interval between fractions for hypofractionated dose regimens [46].

King et al. have also analyzed health-related quality of life (QOL) in patients treated with SBRT (median dose of 36.25 Gy in 4 or 5 fractions). 864 patients from a phase 2 clinical trial of SBRT for localized prostate cancer reported QOL at baseline and at regular intervals up to 6 years. 194 patients remained evaluable at 5 years. It showed, that a transient decline in urinary and bowel domains was observed within the first 3 months after SBRT which returned to baseline status or better within 6 months and remained so beyond 5 years. The same pattern was observed among patients with good versus poor baseline function and was independent of the degree of early toxicities. Thus this demonstrates that prostate SBRT is well tolerated and has little lasting impact on health-related QOL [47].

For intermediate-risk or high-risk prostate cancer, there is even scarcer evidence, as only few trials on SBRT for prostate cancer patients with these features exist. Katz et al. are among those, who treated apart from low- (n = 211), also intermediate- (n = 81) and even high-risk prostate cancer patients (n = 12) with SBRT [48]. 57 of these patients received neoadjuvant hormonal therapy for up to one year. The first 50 patients received a total dose of 35 Gy in 5 fractions with a single dose of 7 Gy that covered at least 96% of the PTV. The subsequent 254 patients received a total dose of 36.25 Gy in 5 fractions with a single dose of 7.25 Gy that covered as well at least 96% of the PTV. The median follow-up of all patients was 60 months (72 months for those with a lower and 60 months for those with a higher overall dose). There was no acute urinary or rectal toxicity Grade III or IV and fewer than 5% of patients experienced any Grade II urinary or rectal toxicities. In regard to late toxicity there were 4% respectively 2% of patients in the 35 Gy group, who experienced urinary/rectal Grade II complications, whereas in the 36.25 Gy group there were 9% respectively 5% patients with urinary/rectal Grade II toxicity and 2% with late Grade III urinary toxicity. 5-year biochemical recurrence-free survival was 97% for low-risk, 90.7% for intermediate-risk and 74.1% for high risk patients. Although the results for high-risk patients in this study are encouraging, one must note that only 12 high-risk patients were included and further data is needed.

In regard of dose escalation Kim et al. were among the first to undertake a dose study of SBRT with 45, 47.5 and 50 Gy in 5 fractions for localized prostate cancer, in which 91 patients were enrolled. At the highest dose level, 6.6% of patients treated developed high-grade rectal toxicity, 5 of whom required colostomy. Grade 3+ delayed rectal toxicity was strongly correlated with volume of rectal wall receiving 50 Gy >3 cm^3^ (p < 0.0001), and treatment of >35% circumference of rectal wall to 39 Gy (p = 0.003). Grade 2+ acute rectal toxicity was significantly correlated with treatment of >50% circumference of rectal wall to 24 Gy (p = 0.010). The authors of this study therefore advise caution when considering high-dose SBRT for treatment of tumors near bowel structures, including prostate cancer and recommend to respect the above mentioned threshold dose constraints in order to minimize risk of severe rectal toxicity [49].

Provided that prostate motion is tracked and accounted for (IGRT), high dose SBRT for prostate cancer may become an attractive option especially for low- and intermediate-risk patients. Regardless of whatsoever risk group, patients who choose SBRT as primary treatment should be enrolled in clinical trials, as the evidence is still very limited.

## Conclusion

There is a growing body of evidence suggesting that prostate cancer has a low α/β-level of 1.4 Gy and therefore lower than that of surrounding organs at risk, such as rectum or bladder. This poses a therapeutic rationale for hypofractionation with the possible result of a better tumor control at a lower toxicity rate. Vital for a safe appliance of hypofractionated schemes are IMRT and IGRT. These tools are the technical prerequisite for administering high single doses. So far there are encouraging results for moderately as well as for higher hypofractionated schemes regardless of the prostate cancer risk group. Nevertheless there are still pending questions and ongoing trials, as well as further follow-up of already conducted trials that need to be awaited, before hypofractionated radiotherapy can be generally recommended. Therefore so far patients who are intended to be treated with a hypofractionated scheme should be enrolled in clinical trials. This is also the case for patients that are to be treated with stereotactic body radiotherapy, which might be even more convenient to patients due to the use of higher single doses and thus shorter treatment time.
